# Supplementary material for: Establishment and characterization of VOA1066 cells: An undifferentiated endometrial carcinoma cell line
Source: PLoS One. 2020 Oct 14;15(10):e0240412. doi: 10.1371/journal.pone.0240412 (PMC7556492; doi:10.1371/journal.pone.0240412)
Supplement: S1 Data — The original western blots were included for Fig 4C, 4E and 4F. (PPTX) [file pone.0240412.s002.pptx]

## Slide 1
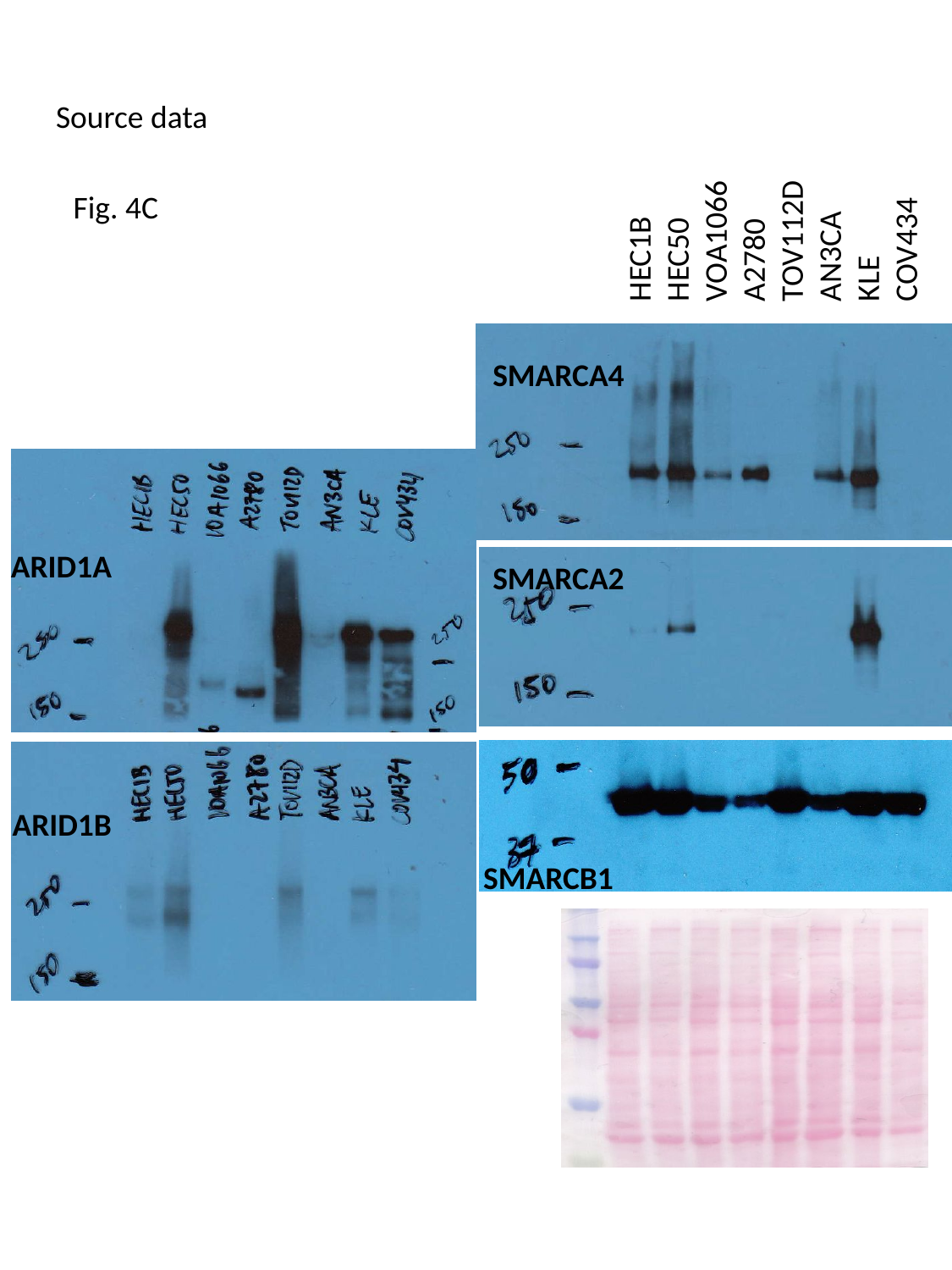

HEC1B
HEC50
VOA1066
A2780
TOV112D
AN3CA
KLE
COV434
Source data
Fig. 4C
SMARCA4
ARID1A
SMARCA2
ARID1B
SMARCB1
20181218

## Slide 2
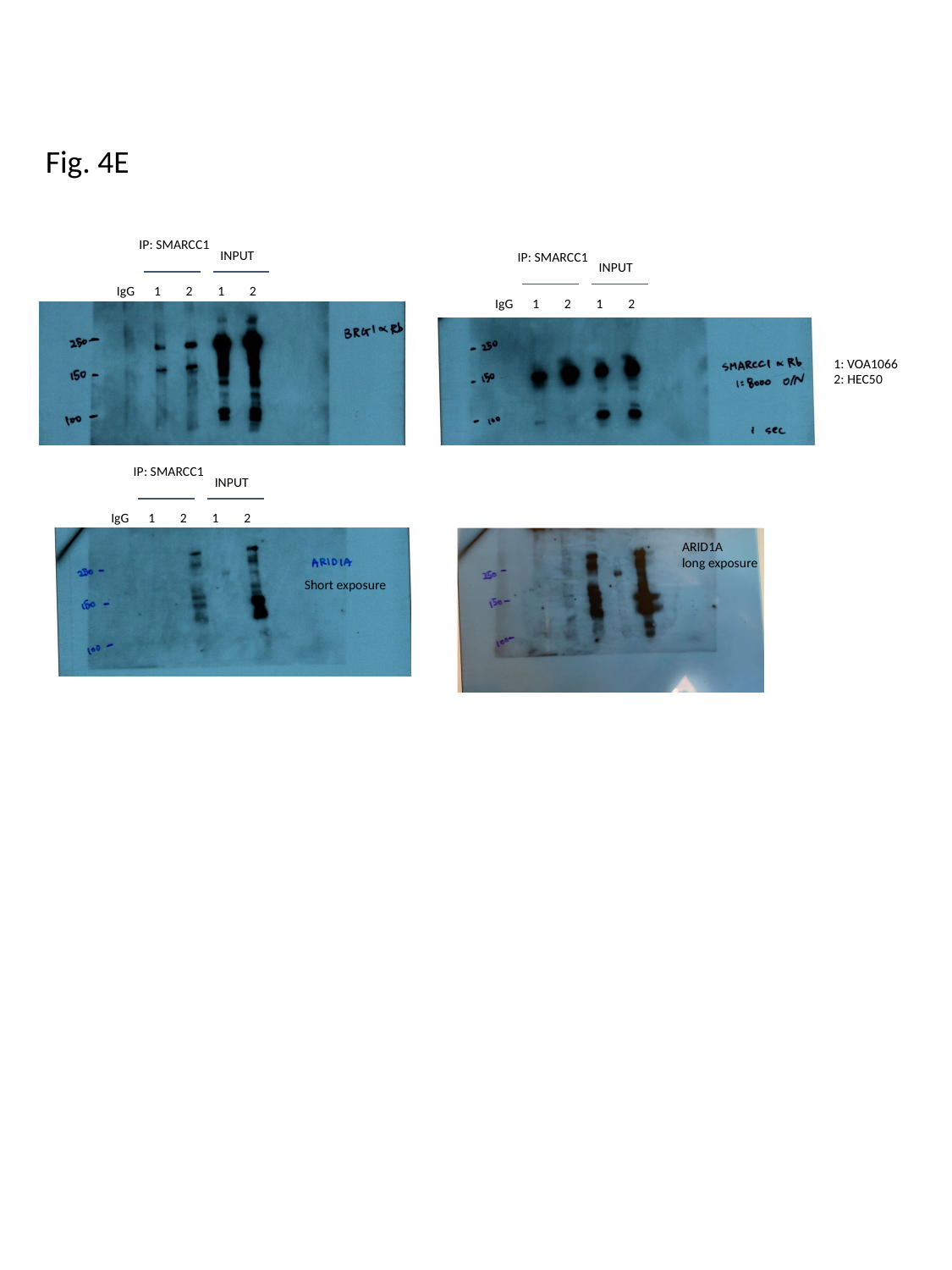

Fig. 4E
IP: SMARCC1
INPUT
IgG
1
2
1
2
IP: SMARCC1
INPUT
IgG
1
2
1
2
1: VOA1066
2: HEC50
IP: SMARCC1
INPUT
IgG
1
2
1
2
ARID1A
long exposure
Short exposure

## Slide 3
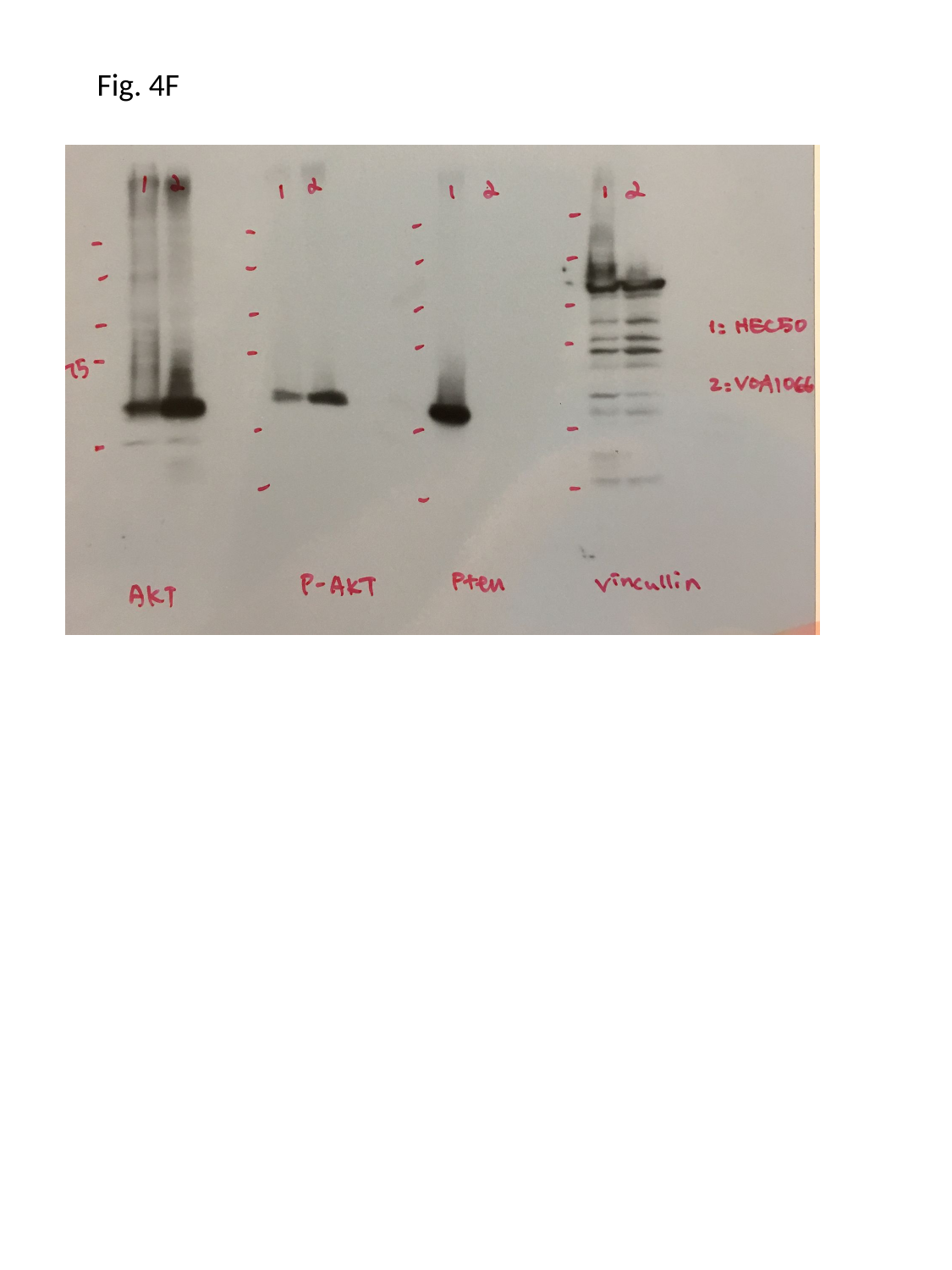

Fig. 4F
