# Supplementary material for: Establishment and characterization of VOA1066 cells: An undifferentiated endometrial carcinoma cell line
Source: PLoS One. 2020 Oct 14;15(10):e0240412. doi: 10.1371/journal.pone.0240412 (PMC7556492; doi:10.1371/journal.pone.0240412)
Supplement: Attachment — Submitted filename: Response to reviewers_0908.docx [file pone.0240412.s003.docx]

**Point by point responses to the reviewers:**

Reviewer #1: Wang et al. describe the generation of an undifferentiated human endometrial carcinoma cell line VOA1066, which harbours characteristics such as intact MMR, ER negativity, genomic stability and is capable of being grown as xenografts. This work is important given the highly aggressive nature of this type of endometrial cancer and the lack of models to investigate the disease. The cell line displays a truncated form of ARID1A, the loss of ARID1B and a p.D1810Y mutation in DICER1, which the authors determine likely retains wild type function. Given the model recapitulates the features of the original tumour from which it was derived, overall the study is well done and establishes a new experimental model that will facilitate future investigation of this rare endometrial cancer type. I have some comments for clarification and suggestions.


Specific points:

1. Figure 4D. Please provide details about the HEC50 cell line in the methods. Please explain the shift in molecular weight for SMARCA4 in the VOA1066 sample. As defects in ARID1A are often associated with constitutive PI3K/AKT pathway activation, the authors should assess pathway activation in addition to PTEN status.

We have provided the information of HEC50 cell line in Materials and Methods section (Lines 115-117). The band shift for SMARCA4 appears to be an artifact, which did not show up in repeated western blot with several more endometrial or ovarian cancer cell lines (figure attached below). Thus, we have replaced the previous SMARCA4 blot with the new one (new **Fig. 4C**).

Since we did not identify any PIK3CA/AKT pathway mutations in VOA1066 cell line, the loss of PTEN expression is likely due to promoter hypermethylation that has been previously reported in endometrial cancer. In agreement with PTEN loss, VOA1066 cells displayed a high level of pAKT at S473 site (Line 299, new **Fig. 4F**), indicating the activation of the PI3K/AKT pathway in VOA1066 cells.

2. The authors speculate that the truncated ARID1A protein will likely result in impaired incorporation into SWI/SNF complexes, but the claim that this model could be used as a model of both defective ARID1A and ARID1B would be improved if this were demonstrated.

Thanks for the great suggestion. We have now performed co-immunoprecipitation in VOA1066 and HEC50 cells. Our data revealed that while SMARCA4 is co-immunoprecipitated with SMARCC1 in both cell lines, only the full length ARID1A protein in HEC50 cells, but not the truncated ARID1A protein in VOA1066 cells, is co-immunoprecipitated with SMARCC1 (new **Fig. 4E**). This finding strongly supports that VOA1066 cell line is defective for both ARID1A and ARID1B. Please refer to Lines 290-296.

3. In addition to the genomic characterisation of the VOA1066 cell line, RNA sequencing analysis would provide important insight into potential pathways that could be targeted clinically and bolster the utility of this line for mechanistic studies.

We agree that the RNAseq analysis will provide insights into potential druggable targets for this disease. However, we believe that the value of performing such analysis on a single cell line or primary tumor is limited. Indeed, we are performing more molecular analyses on a cohort of DDEC samples, including VOA1066, to underpin the molecular pathways underlying the disease development. We believe such study is beyond the scope of this cell line derivation paper.

Typographical errors:
Ethics statement and page 5 - “eithics” should be “ethics.”
Page 9 – “alternations” should be “alterations.”

Thanks for pick up those errors. We have corrected them.

Reviewer #2: DDEC has recently been recognised as a distinct EC subtype for its aggressive behaviour. The mechanism associated with pathogenesis remains largely unknown. No matter how great proportion the lower-grade endometrioid adenocarcinoma accounted for the DDEC, the component of UEC contributed largely to a highly aggressive manner. Therefore, understating of DDEC starts with the knowledge of UEC. This paper established a UEC cell line and validate through specific genomic features. The study is of translational important but needs to address a couple of major points:
1. Molecular signature specific to UEC remains unclear. The cell line established in this paper could be useful to study to role of ARID1A and ARID1B mutations in UEC, but not quite useful for other inactivating mutations such as SMARCA4. Recent studies suggest a role for inactivation of SMARCA4 in UEC (Stewart et al, 2015; Karnezis et al, 2015) but according to the western blotting result of this study, SMARCA4 showed expression in the UEC cell line. It would also be useful to include IHC for SMARCA4.

We have performed IHC for SMARCA4 on the primary tumor and confirmed the intact expression of SMARCA4. Please refer to Line 290-291 (new **Fig 4D**).

2. Grade 2 or 3 endometrioid adenocarcinoma are the 2 most misdiagnoses of DDEC. More markers such as Mullerian epithelial markers: keratin, EMA and PAX8 should be included to validate the portion of UEC in the primary tumor as well as cell line to discriminate from grade 2 or 3 endometrioid adenocarcinoma.

We have performed IHC staining on primary tumor, adjacent normal endocervix tissue and xenografted tumors. Data are summarized in new **Fig 1** and Lines 210-215, which confirmed that VOA1066 primary tumor xenograft tumor lost all 4 common epithelial markers examined.

3. The VOA1066 cell line had more than 30% protein altering mutations compared to the primary tumour, which could be problematic when understanding related mechanisms. It would be helpful to include a functional summary of the cell line specific mutations.

We have included a supplemental table (**S1 Table**), which included detailed mutation information and their predicted impact on function for all somatic exonic non-synonymous mutations that were either shared or private to primary tumor or cell line of VOA1066. We have also calculated the median allelic depths of mutant alleles for either shared and unique mutations, respectively. The average allelic depth for common non-synonymous mutations was 22, while that for non-synonymous mutations unique to either sample was 6. This suggest that the discrepancy between primary tumor and cell line is largely due to the exome sequencing depth, which may not be sufficient to detect rare events in one of the samples. We have added this comment in Lines 276-279.

4. For the IHC of VOA1066 primary tumor, it would be reasonable to include positive controls for ER staining (such as from adjacent endometrioid portions).

We have performed IHC staining on adjacent normal endocervix tissue for all epithelial markers. Data are summarized in new **Fig 1**.
